# Indication-based electronic prescribing intervention reduces unnecessary meropenem use in a safety-net hospital: a quasi-experimental study

**DOI:** 10.1017/ash.2025.10148

**Published:** 2025-11-10

**Authors:** Jannet Manuela Reyna-Quito, Robert Glowacki, Huiyuan Zhang, William E. Trick, Vanessa Sardá

**Affiliations:** 1 Division of Infectious Diseases, Cook County Health, Chicago, IL, USA; 2 Department of Medicine, Rush University Medical Center, Chicago, IL, USA; 3 Health Research & Solutions, Cook County Health, Chicago, IL, USA; 4 Honorary Physician, Cook County Health, Chicago, IL, USA

## Abstract

This quasi-experimental before-and-after intervention study evaluated an indication-based electronic prescribing alert in a 450-bed tertiary care hospital. Implementation reduced meropenem use by 41% without compromising patient safety, demonstrating the effectiveness of this targeted antimicrobial stewardship strategy.

## Introduction

Antibiotic resistance is a major global public health threat, causing approximately 1.27 million deaths each year.^1^ Misuse and overuse of antibiotics accelerate the emergence of multidrug-resistant organisms (MDROs), including extended-spectrum β-lactamase–producing (ESBL) *Enterobacterales* and multidrug-resistant (MDR) *Pseudomonas aeruginosa*, for which carbapenems remain critical last-line therapies.^1^

Time-proven antimicrobial stewardship strategies, such as prospective audit and feedback, are labor intensive and time consuming. To complement traditional stewardship activities, we implemented an indication-based electronic alert for meropenem prescribing as a scalable, system-level intervention that curbs inappropriate carbapenem use, mitigates antimicrobial resistance risk, and maintains patient safety in real-world, resource-limited settings.

## Methods

### Study design and setting

We conducted a single-center, quasi-experimental before-and-after intervention study in a 450-bed tertiary care safety-net hospital. The preintervention period spanned May 2022–April 2023, followed by a 1-month washout and a 12-month intervention period (June 2023–May 2024).

All adult inpatient admissions, except for admissions to the obstetric and pediatric unit, who had at least one meropenem order placed during hospitalization, were included in the study.

### Intervention

An indication-based electronic alert for meropenem orders was implemented in the electronic medical record (EMR). Ertapenem was excluded, as it is rarely prescribed empirically at our institution. Prescribers were required to select one of six options: five predefined, guideline-concordant indications—(1) septic shock; (2) culture-confirmed ESBL *Enterobacterales* or MDR *Pseudomonas aeruginosa* infection; (3) empiric therapy for febrile neutropenia; (4) documented ESBL or MDR *Pseudomonas aeruginosa* infection within the prior six months; and (5) Infectious Diseases service recommendation—or (6) “none of the above.”

If a guideline-concordant indication was selected, the order proceeded without further interruption. However, selecting “none of the above” triggered a conditional interruption—a soft stop in the EMR that temporarily blocked the order unless a valid indication was chosen or the ASP provided case-by-case approval.

The predefined indications were developed by Antimicrobial Stewardship Program (ASP) in accordance with national guidelines and institutional antibiograms, underwent peer review, and were approved by the hospital’s multidisciplinary stewardship committee. No free-text or override option was available, but prescribers could request case-by-case exceptions from the ASP, creating a structured stewardship checkpoint while maintaining clinical flexibility.

### Training session

A one-hour in-person lecture was delivered to Internal and Family Medicine residents during weekly didactics, targeting those responsible for most inpatient meropenem prescribing throughout the hospital, including intensive care units (ICUs). Attendance was expected but not tracked or incentivized. Attending physicians, including surgical specialists, were informed via institutional memo without additional lectures.

### Data collection and outcomes

The primary outcomes were: (a) meropenem utilization (AU), measured as days of therapy (DOT) per 1000 patient bed-days per CDC methodology, and (b) the AU/AR ratio for ESBL-producing *Enterobacterales*.^2^ AR (antimicrobial resistance) represented the incidence of ESBL-producing isolates per 1000 patient bed-days. ESBL events were counted once per patient per admission, defined as the first positive clinical culture from any source. This ratio reflects meropenem use relative to the burden of ESBL *Enterobacterales*, highlighting potential excess empiric therapy compared to the actual occurrence of resistant pathogens.^3^ Secondary outcomes evaluated the clinical impact of blocked meropenem orders, including the incidence of ESBL *Enterobacterales* and MDR *Pseudomonas aeruginosa* isolated from any clinical specimen in affected patients.^4^ Only isolates from the same hospitalization were analyzed to determine whether restricted orders were associated with missed opportunities for appropriate meropenem therapy.

### Analysis

Meropenem use was compared using t tests after confirming normality with the Shapiro-Wilk test. Seasonality was assessed via 12-month additive decomposition. Due to low ESBL incidence, AU/AR ratio analysis was limited to descriptive reporting.

## Results

In the first year, 500 alerts were triggered, with 25% from all ICUs (including medical, surgical and trauma) and 75% from non-ICU settings. Of these, 79% (*n* = 393) met criteria and were approved. The indications included septic shock (53%), ID/ASP team approval (18%), culture-confirmed ESBL *Enterobacterales* or MDR *Pseudomonas aeruginosa* infection (17%), a history of ESBL *Enterobacterales* infection or MDR *Pseudomonas aeruginosa* within the past six months (7%), and empiric treatment for febrile neutropenia (5%).

**Figure 1.**
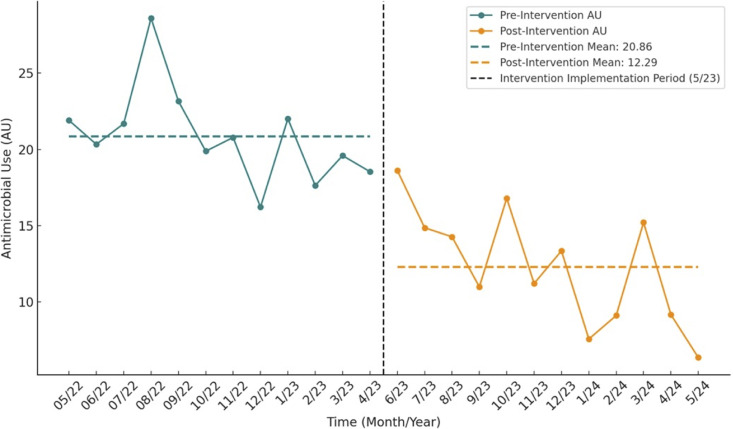
Trend for facilitywide antimicrobial use (AU) for meropenem. AU: Meropenem antibiotic-days per 1000 patient bed-days. *p*-value < 0.001.

Meropenem use declined from 20.86 to 12.29 DOT per 1000 patient bed-days postintervention (*p* < 0.001; 95% CI, 5.39–11.55) (Figure 1). Seasonal decomposition showed minimal variation (SD 2.18; range 7.20; mean 0.00), and 12-month autocorrelation revealed no significant peaks, indicating no annual seasonality.

**Figure 2.**
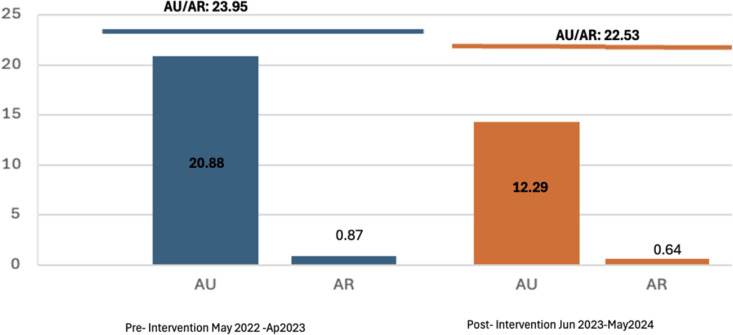
Trend for facilitywide antimicrobial use/antimicrobial resistance (AU/AR) ratio for meropenem/ESBL—producing bacteria events. AU: Meropenem antibiotic-days per 1000 patient bed-days; AR: ESBL-producing bacteria per 1000 patient bed-days; AU/AR: Meropenem use per ESBL- producing bacteria events.

The AU/AR ratio decreased modestly from 23.95 to 22.53, reflecting a decline in empiric meropenem use and a 26.4% relative reduction in ESBL infections (0.87 to 0.64) (Figure 2).

Among the 107 patients (21%) with rejected meropenem orders, no ESBL-producing *Enterobacterales* or MDR *Pseudomonas aeruginosa* isolates were identified that required meropenem therapy during the same hospitalization.

## Discussion

At our institution, the ASP historically relied on restricted-use policies and limited audit-and-feedback to manage broad-spectrum antibiotics. However, meropenem—the sole broad-spectrum carbapenem in our formulary—was prescribed at providers’ discretion, leading to high utilization. This prompted the implementation of the hospital’s first EMR-integrated, indications-based stewardship alert, which provided real-time clinical decision support, point-of-care education, and automated approval to optimize meropenem use while conserving limited stewardship resources.

The intervention achieved a 41.1% reduction in meropenem utilization, and none of the patients with rejected orders (21%) developed ESBL *Enterobacterales* or MDR *Pseudomonas aeruginosa* infections requiring meropenem, suggesting that the alert safely reduced unnecessary meropenem use.

The AU/AR ratio decreased by 1.42, accompanied by a 26.4% decline in ESBL-producing *Enterobacterales* (0.87 to 0.64). While causality cannot be definitively established given the multifactorial nature of resistance trends, these findings are consistent with prior studies demonstrating that stewardship interventions can reduce ESBL incidence over time and support the potential for ASPs to influence resistance patterns longitudinally.^5–7^

Although formal cost-effectiveness was not assessed, the reduction in meropenem use likely translates to resource savings and decreased selection pressure for resistance. Similar EMR-driven initiatives, such as INSPIRE-3, demonstrated that stewardship interventions can reduce antibiotic DOT and associated costs.^8,9^

Routine ASP surveillance also revealed a “squeeze-the-balloon” effect: piperacillin-tazobactam utilization rose from 37.45 to 45.72 DOT per 1000 patient bed-days postintervention, underscoring the need to monitor the broader antibiotic ecosystem during targeted efforts.

Several limitations merit consideration. The 21% rejection rate raises the possibility of “indication drift,” where prescribers may have selected justifying indications rather than strictly adhering to criteria.^10^ and we did not review charts to validate diagnostic concordance. Empiric use for septic shock without prior ESBL risk factors was permitted, which may limit generalizability to institutions with lower MDRO prevalence or stricter stewardship practices. Educational efforts primarily targeted residents, with no engagement of attending physicians and surgical trainees, which may have limited broader adoption.

As a quasi-experimental quality improvement study, we lacked patient-level data, precluding direct comparison of pre- and postintervention populations or adjustment for potential confounders; however equivalent timeframes were chosen to minimize bias.

We acknowledge that culturing practices or patient mix could have influenced the observed reduction in ESBL *Enterobacterales* incidence. However, stable annual culture volumes on institutional antibiograms suggest the decline was not solely due to reduced culture acquisition.

In conclusion, an EMR-integrated, indications-based alert reduced meropenem use and safely avoided unnecessary exposure, demonstrating the effectiveness of automated, real-time stewardship in high-utilization, resource-limited safety-net hospitals.
